# Intervention Mapping to develop a Social Cognitive Theory-based intervention for chronic pain tailored to individuals with HIV

**DOI:** 10.1016/j.conctc.2018.02.004

**Published:** 2018-02-19

**Authors:** Jessica S. Merlin, Sarah R. Young, Mallory O. Johnson, Michael Saag, William Demonte, Robert Kerns, Matthew J. Bair, Stefan Kertesz, Janet M. Turan, Meredith Kilgore, Olivio J. Clay, Dorothy Pekmezi, Susan Davies

**Affiliations:** a Division of General Internal Medicine, University of Pittsburgh, Pittsburgh, PA, United States; b Division of Infectious Diseases, University of Pittsburgh, Pittsburgh, PA, United States; c Department of Medicine, University of California, San Francisco, San Francisco, CA, United States; d Pain Research, Informatics, Multimorbidities and Education (PRIME) Center, VA Connecticut Healthcare System, New Haven, CT, United States; e Departments of Psychiatry, Neurology and Psychology, Yale University, New Haven, CT, United States; f VA HSR&D Center for Health Information and Communication, United States; g Department of Medicine, Division of General Internal Medicine, Indiana University School of Medicine, Indianapolis, IN, United States; h Regenstrief Institute, Indianapolis, IN, United States; i Birmingham VA Medical Center, Birmingham, AL, United States; j Division of Preventive Medicine, Department of Medicine, University of Alabama at Birmingham, Birmingham, AL, United States; k Department of Health Care Organization and Policy, School of Public Health, University of Alabama at Birmingham, Birmingham, AL, United States; l Department of Psychology, College of Arts and Sciences, University of Alabama at Birmingham, Birmingham, AL, United States; m Department of Health Behavior, School of Public Health, University of Alabama at Birmingham, Birmingham, AL, United States

**Keywords:** Chronic pain, HIV, Intervention mapping, Intervention development, Social cognitive theory

## Abstract

Chronic pain is an important comorbidity among individuals with HIV. Behavioral interventions are widely regarded as evidence-based, efficacious non-pharmacologic interventions for chronic pain in the general population. An accepted principle in behavioral science is that theory-based, systematically-developed behavioral interventions tailored to the unique needs of a target population are most likely to be efficacious. Our aim was to use Intervention Mapping to systematically develop a Social Cognitive Theory (SCT)-based intervention for chronic pain tailored to individuals with HIV that will improve pain intensity and pain-related functional impairment. Our Intervention Mapping process was informed by qualitative inquiry of 24 patients and seven providers in an HIV primary care clinic. The resulting intervention includes group and one-on-one sessions and peer and staff interventionists. We also developed a conceptual framework that integrates our qualitative findings with SCT-based theoretical constructs. Using this conceptual framework as a guide, our future work will investigate the intervention's impact on chronic pain outcomes, as well as our hypothesized proximal mediators of the intervention's effect.

## 1 Introduction

Chronic pain is defined as pain lasting for more than three months, beyond the period of normal tissue healing [1]. Examples of chronic pain include regional musculoskeletal pain (e.g., low back pain, knee pain), widespread pain including fibromyalgia, headaches, and peripheral neuropathy. Chronic pain is an important public health problem. Recent studies suggest it occurs in approximately 15% of individuals in the general population [2,3] and can be associated with significant disability [4,5].

Chronic pain is an important comorbidity among individuals with HIV. For reasons that are not fully understood, chronic pain occurs in as many as 30–85% of these patients [6,7]. In individuals with HIV, chronic pain is associated with up to 10 times greater odds of functional impairment [8], and can also be associated with suboptimal retention in HIV primary care [7]. The recently-released Department of Health and Human Services National Pain Strategy identified chronic pain in vulnerable populations, including individuals with HIV, as a priority area of investigation [9].

Management of chronic pain has typically included both pharmacologic and non-pharmacologic therapies. A mainstay of pharmacologic treatment for chronic pain has been long-term opioid therapy. However, in the past few years, the risks of opioids including addiction and overdose have been increasingly recognized [10]. Individuals with HIV are more commonly prescribed opioids than individuals in the general population [11], despite an apparent susceptibility to opioids' mortality risk [12] and drug-drug interactions with antiretrovirals [13]. Therefore, development of non-pharmacologic approaches for individuals with HIV is of particular importance.

Behavioral interventions are widely regarded as evidence-based, efficacious non-pharmacologic interventions for chronic pain in the general population [9]. An accepted principle in behavioral science is that theory-based, systematically-developed behavioral interventions tailored to the unique needs of a target population are more likely to be efficacious [14]. The present study emerged from evidence that such an intervention has not yet been developed for chronic pain in individuals with HIV. Our recent systematic review of existing chronic pain interventions in individuals with HIV included all interventions for chronic pain, both pharmacologic and non-pharmacologic, that have been tested in individuals with HIV [15]. Eleven interventions met the inclusion criteria, only two of which were behavioral interventions [16,17]. Neither of these interventions was developed based on a behavior change theory, and neither was tailored to individuals with HIV in a systematic way. Both interventions suffered from poor adherence, reported only small effect sizes, and have not been studied further.

Therefore, our aim was to systematically develop a theory-based intervention for chronic pain tailored to individuals with HIV that will improve key chronic pain outcomes: pain and pain-related functional impairment, including physical and emotional function [18].

## 2 Methods

Intervention Mapping (IM) is a stepwise process for the systematic development and evaluation of a theory- and evidence-based behavioral intervention that is tailored to the target population [19]. Here, we present our approach to the first four steps of IM: 1) needs assessment, 2) identification of behavioral targets and creation of a behavior change matrix using Social Cognitive Theory (SCT), 3) intervention design, and 4) intervention production. Steps 5 and 6, program implementation and evaluation, are the next steps in our research program.

The IM process – especially Steps 2 through 4 – is informed by qualitative inquiry. For this purpose, we recruited 24 patients from a large HIV clinic in the Southeastern US to participate in 12 in-depth individual interviews followed by three focus groups. The objective was to use patient ideas and preferences to inform our approach to Steps 2 through 4. To analyze our data, we used an independent, thematic approach with three coders (JSM, WA, SRY). Initial discussions led to agreement on key themes and generation of a codebook, which one investigator (SRY) used to code the remaining transcripts. Subsequently, we conducted seven in-depth interviews of HIV clinic providers, including some who hold leadership positions. We recruited providers across disciplines including physicians, nurse practitioners, nurses, and a pharmacist, and approached the data using the same design and analytic techniques [20]. Preliminary results from patient interviews and focus groups have been published previously, and suggested the importance of a group setting, peer interventionists, and groups limited to HIV + participants [20]. How this work and additional results of these qualitative investigations were integrated into the IM process is detailed here in the description of each IM step.

### 2.1 Intervention mapping steps

*Step 1:* The first step of IM is to conduct a needs assessment of the problem in the target population. The results of this step were described briefly in the introduction and synthesized in a systematic review [15]. We concluded that a behavioral intervention for chronic pain tailored to individuals with HIV is needed.

*Step 2:* The second step of IM is to create “change objectives.” Change objectives are actions aimed at changing key behaviors that influence the desired outcomes. Each change objective will become an intervention session.

We identified key change objectives from the robust literature on evidence-based Pain Self-Management (PSM) interventions [[21], [22], [23], [24]]. PSM interventions draw from cognitive-behavioral therapy, and are manualized interventions designed to reduce pain intensity and pain-related functional impairment in the general population. They can be delivered by a variety of health care professionals (nurses, psychologists, and social workers) trained on the PSM protocol, making them well-suited for diverse settings. PSM interventions provide pain education, and also target patient-centered self-management of key behaviors (e.g., physical activity). These behaviors are each directly targeted by a change objective (e.g. increase physical activity). PSM interventions were named by the National Pain Strategy as evidence-based, scalable approaches to chronic pain management that can be tailored to the needs of specific populations [9].

We selected a specific PSM intervention as a starting point for our work. This intervention is called Stepped Care for Affective disorders and Musculoskeletal Pain (SCAMP) [25,26]. We chose SCAMP for several reasons. SCAMP was initially developed as a PSM intervention for patients with chronic musculoskeletal pain and depression, a common comorbidity in HIV. SCAMP was also delivered in primary care settings, as we envision for our intervention. Interventionists were nurse care managers, who are more readily available in HIV care settings than psychologists. SCAMP was delivered as a 12-week intervention consisting of one-on-one sessions with a staff interventionist. Each session addressed a unique PSM behavior/change objective (e.g., physical activity, thinking differently about pain, stress management, alternative therapies, talking to your doctor/nurse about pain, utilizing community resources).

As previously described [20], we collected qualitative data (initially interviews, then focus groups) from HIV-infected individuals with chronic pain recruited from an HIV outpatient clinic in the US. Participants who had at least moderate pain for more than three months met eligibility criteria, and we purposively sampled individuals with symptoms of depression or anxiety and current substance. During in-depth individual interviews, we began by asking participants to discuss potential behaviors/change objectives they would like to include in the intervention. Then, they were asked to review the SCAMP manual. Participants were asked to provide feedback regarding the inclusion of each session (behavior/change objective) in the intervention and to suggest new topics.

Next, we completed three focus groups: one of interview participants, and two of new study participants. We conducted a card sort exercise of all potential session topics. Sessions assessed included all SCAMP sessions plus potential session topics that emerged from interview participants (improving mental health, losing weight, sleeping better, taking chronic pain medications, building self-worth, meditation, addressing addiction, improving posture, and distraction). Participants were asked to identify the five most important and three least important sessions, in order. Using a mixture of card sort data, additional qualitative data, and expert opinion, we identified the ten sessions most salient to our intended population.

*Step 3:* The third step of IM is intervention design. This includes making important choices about the intervention's structure based on prior knowledge of the target population – in this case, our qualitative work. Additionally, a fundamental premise of IM is that all intervention components have theoretical underpinnings. Therefore, Step 3 also includes systematic integration of theory throughout the intervention.

We selected Social Cognitive Theory (SCT) as it is a widely-cited foundation for chronic pain behavioral interventions [27]. This decision has face validity: SCT is a learning theory, and posits that even in the face of stressors (e.g., pain), people can learn to change their behavior (e.g., engage in regular physical activity) through a variety of methods informed by key theoretically-informed constructs (e.g., self-efficacy, outcome expectations, self-regulation). Table 1 describes key SCT constructs.

**Table 1:** Social cognitive theory constructs.

Construct	Description
Observational learning	Learning can occur by observing others. People are most likely to pay attention if the information is perceived as valuable and if it is delivered in a way that is understandable.
Self-efficacy	Self-efficacy is the person's belief in their ability to successfully complete the task. The four key ways to develop or increase-self efficacy are mastery experience (prior experience that an individual can draw from), social modeling, improving physical and emotional states, and verbal persuasion.
Outcome expectations	Outcome expectations are the beliefs that something good will come from participating in the intervention; the outcome must be something that is perceived as important. Social outcome expectations are how others evaluate one's behavior, and whether this is viewed as important or not. Self-evaluative outcome expectations are anticipation of how one will feel about themselves if they successfully complete the intervention.
Self-regulation	Self-regulation is the willingness to perform a new behavior now to reach a goal in the future. This is not achieved through sheer willpower or brute force, but rather through gaining skills. There are six ways to achieve self-regulation: self-monitoring (observing or recording one's behavior in a systematic way); goal setting; feedback from others; self-reward; self-instruction (talking oneself through a behavior); and enlistment of social support.

How people go about changing their behavior – in other words, the practical application of the theory to address each change objective identified in Step 2 – can be informed by qualitative inquiry. The Results of Step 3 presented below include the theoretical constructs alongside proposed practical applications, and their supporting qualitative results. These practical applications apply across change objectives.

*Step 4:* The fourth step of IM is intervention production, or the creation of each intervention component. A member of our team (WD) is a pain psychologist and an experienced developer of low-literacy chronic pain intervention manuals. The Principal Investigator (JSM) and WD collaboratively wrote the participant manual. Written materials were based on the SCAMP manual and an evidence-based low-literacy manual (Learning About Managing Pain or LAMP), which WD has delivered in prior studies [28]. With the permission of these manual authors, we used existing passages verbatim when appropriate, given that they are already tested, which serves to strengthen the manual that we produce. However, we created new content when no appropriate content existed. To elicit feedback, we conducted one “pre-testing” focus group of 9 participants during which we presented the overall intervention structure and the manual content. After this focus group, we conducted a final round of revisions.

### 2.2 Conceptual framework development

**Fig. 1:**
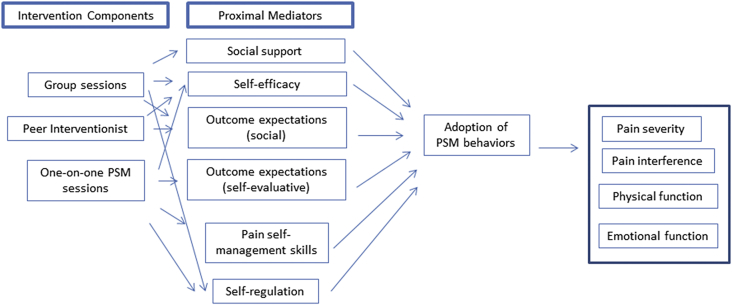
Conceptual framework for a pain self-management (PSM) intervention tailored to individuals with HIV.

The IM process provided a preliminary understanding of how our intervention may work to improve pain and pain-related functional impairment [29]. This allowed us to develop a SCT-based conceptual framework to pictorially represent our intervention's proposed mechanism (Fig. 1).

This study was approved by the Institutional Review Board of the University of Alabama at Birmingham.

## 3 Results

**Fig. 2:**
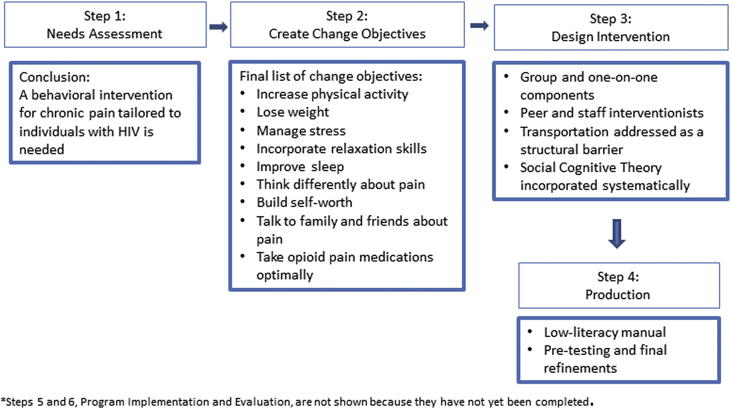
Intervention mapping process and results*. *Steps 5 and 6, program implementation and evaluation, are not shown because they have not been completed.

Here, we present the results of IM Steps 2–4, which are informed by our qualitative work, and explain how we made key intervention decisions. This process is summarized in Fig. 2.

Of the 24 patient participants in interviews and focus groups, most were male (17) and African American (19). Mean age was 48 years (range 33–68), and nearly all (23) participants had an undetectable viral load. Mean pain severity “on average” on the Brief Pain Inventory was 6.6 (scale 0–10), and mean interference was 7.2/10 (missing = 4). Of seven providers, five were at least 50% devoted to clinical activities, and three held clinic leadership positions.

### 3.1 Intervention sessions

Most patient participants believed that pain education should be a required session. Additionally, participants were open to including all other SCAMP sessions. In the card sort exercise, results varied between focus groups, but posture and meditation were always at or near the least preferred. Therefore, these two topics were eliminated. Among the topics that remained, mental health care was incorporated into the pain education session, distraction was incorporated into relaxation skills and stress management, and opioid addiction was incorporated into a chapter on taking opioids (rather than chronic pain medications broadly). We developed the remaining chapters (weight loss, sleep) as they often ranked near the top. In addition to pain education, the final list of the remaining nine change objectives/intervention sessions is included in Fig. 2.

### 3.2 Intervention structure

Our qualitative work revealed several key patient and provider insights that guided the intervention's design. These are as follows:

aOne-on-one intervention sessions

One theme that emerged was participants' desire to keep the content relevant, and tailored when possible. For example:

*Unidentified male: As long as you are giving people good, heartfelt information, and stuff that they can actually use, they will always come back. If I feel like you are giving me junk, I am not coming back. (Patient)*

We agreed with participants (see Step 2 above) that a pain education session should be offered to everyone. This session is essential to understanding subsequent sessions. To meet participants' preference for tailoring, we allowed participants to choose five of the remaining nine behavioral target sessions. To best achieve tailoring, we chose a one-on-one session format for content delivery.

Some participants voiced the importance of certain content being delivered by a clinical expert. For example:

*I want them to be able to tell me … just not tell me … Oh, I'm sorry to hear for your pain. I know that really hurts … I need them to be able to say …. Do X, Y, Z and this is what is going to be done for you. I don't think the average person volunteer off the street is going to be able to do that. I think they need to be a trained counselor or whatever. (Patient)*

Therefore, we decided that the one-on-one PSM sessions would be delivered by a staff interventionist, who we will call a “pain coach.” Due to scalability concerns, we identified staff commonly found in HIV clinics to serve as pain coaches. Rather than selecting a clinical psychologist as is sometimes done in behavioral interventions, we opted for master's level staff (e.g., social worker, health educator, nurse case manager). This is also consistent with SCAMP's approach.

b Group and peer components

Our previously-published qualitative results found that participants strongly preferred a group component to foster social support for their chronic pain. They also preferred groups that only included HIV + persons to reduce stigma and protect participant confidentiality about HIV status. Finally, they preferred the involvement of a peer leader to learn how others successfully navigate living with both HIV and chronic pain [20]. We will call this peer a “pain pal,” a shorthand title suggested by one of our patient participants. To have skill-based content delivered by an “expert,” we decided that the pain pals will facilitate the group sessions together with the pain coaches.

Participant feedback supported SCAMP's structure of 12 weekly sessions, so we incorporated that into our intervention. To incorporate the group component, we structured our intervention by alternating one-on-one PSM sessions and group sessions, so that participants would receive six of each.

c Transportation vouchers

Transportation was identified as a major structural barrier to participation. Therefore, we decided to assess this barrier routinely at the beginning and perioidically throughout the intervention, and provide transportation vouchers whenever needed.

### 3.3 Incorporating theory

We identified practical applications of each theoretical construct that could be applied broadly, across change objectives. For example, self-monitoring is an important construct that influences self-regulation, a key component of SCT. Our practical application of self-monitoring is for participants to complete “homework and tracking” sections of the manual for each one-on-one session either at home or with the interventionist. This practical application is supported by our qualitative results. Our team employed this process across all SCT constructs, as summarized in Tables 2a and 2d.

**Table 2:** Incorporation of theoretical constructs.

a: Self-regulation		
Six constructs that contribute to self-regulation:	Practical application(s)	Supportive qualitative results (quotes)
Self-monitoring	-Complete “homework and tracking” section of the manual for each one-on-one session either at home or with interventionist	You have two options there. One, an open mind, an open discussion. And the other one is a – give me some – take these with you. Look over and read it. Bring your ideas back to me like homework in that aspect, you know. And we'll discuss this then and we'll say what we're going to talk about. You can take this home with you and you got your time to sit there and go through it. And when we come back and we meet again, we'll discuss these things.” (Patient)
Goal-setting	- Set long-term goals (what the participant could achieve if pain was better controlled) during first one-on-one session. - Set specific short term pain-related goals at each one-on-one session. (e.g., take a 15-min walk this week)	“I will get into saying like right here you got short-term activities and long-term activities. The long – the short term is something like we can come in as we're sitting here. And we'll discuss it right quick. Long term is something like a homework thing. You go home and we write out this thing and we're going to give it a try so when we have our next meeting we'll come back, and see why we came out with it. You tell us how we came out with it, what we did. That would be definitely in my program. That would definitely be with my group.” (Patient)
Feedback from others	- Share progress on goals with the group. - Listen to and incorporate feedback received at group and individual sessions. - Brainstorm solutions to pain-related problems in the group.	“[S]ay for instance you come in that day and you have a peer coach that day. You are giving your demonstration. Well, you start to talk. As you begin to talk and go into your demonstration, you begin to explain how this happened and how that unfolded and that unfolded. Then you go into how you remedied it, how you got a remedy for that. Well, you are giving me a whole package, not just what happened, but what was the outcome on what you did to overcome what was happening to you. I do not want to know just that you had these symptoms, but what did you do to relieve these symptoms?” (Patient)
Self-instruction	- Participants will write down how they will achieve their weekly goal; they can refer back to this throughout the week when they get stuck.	“you could do that from the time you wake up till it's time to go to bed what did you do today to reduce your pain or whatever have you? Was your pain able to be reduced? Don't forget to put on there where did you have pain, you know make us up a little notebook or whatever have you. I keep up with stuff like that and I try to write it myself or remember it in my head. And when we come to our sessions you have your notebook right there so you don't have to try to remember and when the question is asked you have the information right there. Also at the bottom you put “My goal is to such and such and such”. You might change your goal over the course of three or four weeks, but that's okay you got to let your people know that's okay. You also have to let them know that if they didn't do it, move on to the next day or whatever have you. So being held accountable is going to be easier and getting more results than you just turn us loose and I see you all next week.” (Patient)
Enlistment of social support	- Participants will engage in >80% of group sessions. - Group sessions will offer a chance for follow-up and accountability on individual goals.	“To me it is more like you're dedicated for yourself. I mean you the one in pain, so you make the pledge to yourself and then to your peers because if you see them doing it, you're more apt to do it. If you see it working for them then you'll be more apt to do it, that's the way I see it. It's like a goal or something.” (Patient)
**b: Self-efficacy**		
**Four constructs that contribute to self-efficacy:**	**Practical application(s)**	**Supportive qualitative results (quotes)**
Mastery experience	- Practice each PSM skill (e.g., physical activity, thinking differently) at a frequency determined by the participant - Continue to practice each PSM skill for the duration of the intervention, not just for the week after that skill's session is delivered	“I would go down my list and like I said, teach a person to do [a skill] the day before, not two or three days before. I think it's fresher and on your mind if you do it the night before and it kind of keeps in perspective what's important … And I think that should be a homework assignment like every week from – or at least every day when you come in next week to the session you need to have your five to do lists for five weekdays that you did the night before that day, you know? Be a part of the class. Just to get them started, to teach them thinking about how to do their lists and stuff like that. I think it can be taught. And if they keep it simple for most important to least important hey, three or five things. And then if you get that done, if you want to add something else and you're capable physically of doing something else, hey do it. You know, just a little practice. A little practice.” (Patient)
Improving physical or emotional states	Making sure that participants are in the best possible physical and emotional state before every session (e.g., brief deep breathing or mediation exercise before sessions begin)	“No, um you know there could be different classes. Music was just one of them. I think meditation classes, people need to be taught how to meditate appropriately, how to center themselves, how to learn to relax, what helps them relax. Um, you know just learning about um, I guess taking care of the body like mentally and different things you can do to you know slow your blood pressure down, slow your heart rate and things like that. I think a lot of it is going to have to learning to relax. But I think we tend to be so over the top because we're hurting all the time that we can just kind of get lost in the frenzy of it all. So I think a lot of people don't really know how to meditate, how to relax, you know? Even art therapy, you know painting and writing and things like that. I think a lot of that would be beneficial. (Patient)
Verbal persuasion	Cheerleading from peers, staff interventionists, and other intervention participants	**“**For a person, I'd put it like that for a person that if a person tries to lift with weight and he sees a person that they're going to go help them lose weight in a good way, then they'll be motivated to do it. You know, you can encourage that person. (Patient)
Social modeling	Pain pals and group sessions: showing the participant that others like themselves can achieve important pain-related goals	*Because the peer coach, if he or she has experienced some of the things that you are going through, then they can say, “I did this when this happened to me. I experience this when this happened to me. Mentally, I was feeling this way, so I had to do this. Physically, I had to do this because I was feeling this way.”* (Patient) *And especially if someone, one of their peers can give them some strategies to – when this happens this is what I do or I've come through it, I struggled just like you did and now I'm on the other side, there's hope. Now I think that can instill hope with them. It can instill like I said a sense of community, I think that's really important so I think these are – this is great patient feedback.* (Provider)
**c. Observational learning**		
**SCT construct**	**Practical application(s)**	**Supportive qualitative results (quotes)**
Observational learning	Pain pals and group sessions: use to allow participants to observe others' successes	*“Because I don't know everything. I know what works for me sometimes but other than that sometimes I sit at home and why is this happening today, why do I feel like this today? What is going on, I did everything I was supposed to do yesterday and it's different. So that's how a session to me should run and it's not just me talking, it's everybody having conversation to help everybody else.”* (Patient
**d. Outcome expectations**		
**SCT construct**	**Practical application(s)**	**Supportive qualitative results (quotes)**
Social outcome expectations	Pain pals and group sessions: use to review which session each participant attended, goal-setting, and how they are using the intervention. This will set a social outcome expectation to attend sessions and work on goals.	*Yeah. A pat on the back, even if you have to – realizing that this person you can't do anything to please this person, this, that and the other and nothing is working and that special attention. I do more for people when I get special attention. And I'm serious, we all want to do better, we all want to help but you have to find a way to get to that person and you get to the point where you start to care about people in your group. And so you go home yourself and you put up some stuff and I mean you talked to this person when you all come back to the group and say “Hey I read this, that and the other” and I tried it and it worked. And it might be something that the peer counselor or the care manager didn't see. So everybody has a part.* (Patient) *Me being responsible to my group or whatever have you is – it gives me something to do. It gives me a reason to keep on pushing so I can say this is working.* (Patient) *When you don't have family or a group like this would become your family a lot of times you – and you're accountable to somebody and it feels like somebody cares like you're doing something good for the benefit of the people whereas if you don't have a family and then you're going through what you're going through and you're likely to drill into another area, just that you know being accountable in a group setting I think it would just make people who don't have that in their life.* (Patient)
Self-evaluative outcome expectations	One-on-one sessions: encourage participants to complete goals and think about how they will feel when they do.	*Two is how many floors, how many steps can I go up before I'll be distracted or I continue to do what I got to do? And then see, these are goals that I'm setting in my mind to get away from my pain. And I'm going to go down, I got – we got four flights of steps here. Can I make two? Can I make one flight? Can I make two flights? Okay. This is what I'm doing. But I'm in pain now. But I'm going up and down the steps. And my mind is not on the pain but it's where I'm going.* (Patient)

### 3.4 Manual creation

Our manual consists of approximately 50% completely new content, and 50% content derived from the two interventions mentioned previously (SCAMP and LAMP). The final version of the manual is written at below a 6th grade level (Flesch-Kinkaid 2.4, Gunning-Fog 5.6) to address health literacy challenges. We produced the manual in collaboration with a graphic designer, using illustrations to augment the text. Table 3 lists the session topics and their contents.

**Table 3:** One-on-one session topics.

Topic	Session contents^a^
Introduction to your chronic pain	Introductions, pick your sessions, learning more about chronic pain, gate control theory, chronic pain and emotions
Physical activity and your pain	What is physical activity, pros and cons of physical activity, how you spend your days, physical activities you enjoy, pacing
Losing weight to improve your pain	Weight loss and pain, your weight loss picture, how to change how we eat
Stress management and your pain	Introduction to stress, how stress affects you, your experience with stress, stress and pain, managing stress
Relaxation skills to prevent your pain	Relaxation and pain, deep breathing, progressive muscle relaxation, visualization, mindfulness
Sleeping better to help your pain	Importance of sleep, relationship between sleep and pain, things that can hurt sleep, general ways of helping sleep issues
Thinking differently about your pain	Unhelpful thoughts, working to change our unhelpful thoughts
Building self-worth	Unhelpful beliefs, working to change our unhelpful beliefs
Talking with your family and friends about pain	Talking about our pain, ways of talking with others, talking with healthcare providers
Taking opioid pain medications	Your pain medicines, how opioid pain medications work, what the research shows about opioids, taking opioids the way they are prescribed, using strategies besides opioids

a All sessions include goal-setting and homework.

### 3.5 Pre-testing

The most significant clarification from the pre-testing process was the role of the peer (pain pal). In our prior qualitative work, we identified co-leadership by a peer, and sponsor or mentor as being important. This focus group clarified that rather than having a singular sponsor like in 12-step programs who could be called upon between sessions for help, participants should be encouraged to contact each other and the pain pal.

### 3.6 Conceptual framework

Based on the work described above, we present an SCT-based conceptual framework for our intervention's mechanisms (Fig. 1). We posit that the three intervention components – the group sessions, peer involvement (“pain pal”), and the one-on-one sessions – will influence proximal mediators of the intervention's effect. These proximal mediators are the theoretical constructs discussed here. As our previous work emphasizes the importance of social support for management of chronic pain, it is included as an important proximal mediator. PSM skills are also included, as skill acquisition is an essential part of behavior change. The arrows connecting intervention components with proximal mediators are based on the information described in Tables 2a and 2d For example, social support is derived primarily through group sessions, while self-efficacy includes social modeling by pain pals, self-monitoring in individual sessions, and feedback during group sessions. These proximal mediators then lead to the adoption of PSM behaviors, which lead to improvements in pain-related outcomes.

## 4 Discussion

To our knowledge, this is the first research to systematically develop a theory-based intervention for chronic pain that is tailored to the needs of individuals with HIV. While many interventions report a theoretical basis, IM allowed us to methodically integrate SCT throughout every aspect of this novel intervention. Additionally, our formative qualitative work tailored the intervention to our population's unique needs and preferences. We assert that our rigorous intervention development process maximizes the intervention's likelihood of efficacy, which we will investigate in future studies.

Our previous qualitative work reported patient preferences for group sessions and peer involvement [20]. However, the IM steps used theory to operationalize these intervention components, and helped us understand how their inclusion will influence proximal mediators of the interventions' effect. The resulting conceptual framework will serve as a roadmap for IM Steps 5 and 6. For example, it will help us devise an evaluation plan to assess potential mediators and moderators of the intervention's effects. Once the intervention mechanisms of action are more fully understood, future versions of the intervention may augment or omit certain components in the implementation phase.

Our approach has limitations. Our intervention is tailored to address chronic pain in individuals with HIV, and thus its applicability beyond this population was not addressed. Additionally, we conducted our formative qualitative work at a single HIV clinic in the Deep South. It is possible that additional regional or clinic-specific tailoring may be necessary during implementation/dissemination. However, we assert that individuals with HIV and HIV treatment settings are more similar than they are different, and that fundamental concerns emergent in our qualitative work and IM exercise are likely to apply broadly.

## 5 Conclusions

In sum, we have developed an intervention that has a high likelihood of acceptability and efficacy when tested in future studies. Our formative IM work will serve as a foundation for our future studies that investigate this intervention's efficacy and implementation/dissemination. Additionally, few studies detailing the process of integrating theory into an intervention have been published. We hope that our approach can guide others seeking to use IM to develop behavioral interventions.

## Funding

This work was supported by the National Institutes of Health (K23MH104073 to JSM, K24DA037034 to MOJ); the University of Alabama at Birmingham (UAB) Center for AIDS Research, a National Institutes of Health funded program (P30 A1027767) that was made possible by the following institutes: National Institute of Allergy and Infectious Diseases, National Cancer Institute, National Institute of Child Health and Human Development, National Heart, Lung, and Blood Institute, National Institute of Mental Health National Institute on Aging, Fogarty International Center, and Office of AIDS Research; and the PRIME VA Health Services Research Center (CIN 13–047).

## Conflicts of interest

The authors have no competing interests to declare.
